# Procyanidins attenuate neuropathic pain by suppressing matrix metalloproteinase-9/2

**DOI:** 10.1186/s12974-018-1182-9

**Published:** 2018-06-21

**Authors:** Cailong Pan, Chaoyu Wang, Li Zhang, Ling Song, Yuan Chen, Bingqian Liu, Wen-Tao Liu, Liang Hu, Yinbing Pan

**Affiliations:** 1 Neuroprotective Drug Discovery Key Laboratory of Nanjing Medical University, Department of Pharmacology, Nanjing Medical University, Nanjing, 211166 Jiangsu China; 2 Jiangsu Key Laboratory of Neurodegeneration, Department of Pharmacology, Nanjing Medical University, Nanjing, 210029 China; 3 Department of Anesthesiology, Nanjing First Hospital, Nanjing Medical University, Nanjing, 210006 China; 4 Department of Anesthesiology, The First Affiliated Hospital of Nanjing Medical University, Nanjing, 210029 China; 5 Department of Anesthesiology, Children’s Hospital of Nanjing Medical University, Nanjing, 210029 China; 6 Department of ophthalmology, The First Affiliated Hospital of Nanjing Medical University, Nanjing, 210029 China; 7 Neuroprotective Drug Discovery Key Laboratory of Nanjing Medical University, Department of Pharmacology, Nanjing Medical University, Nanjing, 210029 China

**Keywords:** Neuropathic pain, MMP-9/2, Interleukin-1β, Procyanidins

## Abstract

**Background:**

Management of neuropathic pain is a real clinical challenge. Despite intense investigation, the mechanisms of neuropathic pain remain substantially unidentified. Matrix metalloproteinase (MMP)-9 and MMP-2 have been reported to contribute to the development and maintenance of neuropathic pain. Therefore, inhibition of MMP-9/2 may provide a novel therapeutic approach for the treatment of neuropathic pain. In this study, we aim to investigate the effect of procyanidins (PC), clinically used health product, on MMP-9/2 in neuropathic pain.

**Methods:**

The nociception was assessed by measuring the incidence of foot withdrawal in response to mechanical indentation in mice. Cell signaling was assayed using gelatin zymography, western blotting, and immunohistochemistry. The BV2 cells were cultured to investigate the effects of PC on microglia.

**Results:**

Both in vitro and in vivo administration of PC significantly suppresses the activity of MMP-9/2*.* Oral administration of PC relieves neuropathic pain behaviors induced by chronic constriction sciatic nerve injury (CCI) in mice. Additionally, PC blocks the maturation of interleukin-1β, which is a critical substrate of MMPs, and markedly suppresses CCI-induced MAPK phosphorylation and neuronal and microglia activation, including the reduced phosphorylation of protein kinase C γ and NMDAR1. Furthermore, PC decreases the phosphorylation of p38 mitogen-activated protein kinase and inhibits the translocation of nuclear factor-κB (NF-κB) in microglia.

**Conclusions:**

PC is an effective and safe approach to alleviate neuropathic pain via a powerful inhibition on the activation of MMP-9/2.

**Electronic supplementary material:**

The online version of this article (10.1186/s12974-018-1182-9) contains supplementary material, which is available to authorized users.

## Background

Neuropathic pain is a major health concern that represents a considerable social and economic burden worldwide. Effective treatment, however, is hampered by an incomplete understanding of neuropathic pain’s pathogenesis [1]. A growing body of evidence suggests that neuroinflammation plays a vital role in the pathogenesis of neuropathic pain. Central nervous system (CNS) neurons and microglia, as well as pro-inflammatory cytokines secreted by these cells, have all been implicated [2–4].

It is generally believed that proinflammatory cytokines released by microglia, including interleukin (IL)-1β, tumor necrosis factor (TNF)-α, and IL-6, enhance the hyperactivity of dorsal horn neurons, which cause the central sensitization [5]. Notably, IL-1β is the most important factor in inflammatory processes, consisting of precursor form and mature form. Although caspase-1, also known as IL-1β-converting enzyme, is a well-documented protease for IL-1β activation, other enzymes such as MMP-9 and MMP-2 are also implicated in IL-1β cleavage. Study shows that nerve injury-induced spontaneous discharge in sensory neurons releases MMP-9 and pro-IL-1β to the extracellular matrix, where MMP-9 cleaves pro-IL-1β to produce active IL-1β, which then acts on adjacent nociceptive neurons to produce hyperexcitability [6].

Matrix metalloproteinases (MMPs) belong to a large family of zinc-dependent endopeptidases that play a critical role in neuroinflammation through the cleavage of extracellular matrix proteins, cytokines, and chemokines. In particular, recent studies have demonstrated that matrix metalloproteinase (MMP)-9 and MMP-2 contribute to neuropathic pain following nerve injury [7]. MMP-9/2 also enhance neuronal transmission by phosphorylation of *N*-methyl-d-aspartate receptor (NR)1 and NR2B in neurons [8]. But a safe and effective MMP-9/2 inhibitor that can be used in clinic for the treatment of neuropathic pain is still not available. Thus, we are encouraged to search for a safe and effective natural product to manage neuropathic pain via an inhibition on MMP-9/2.

Previous studies suggest that MMP-9/2 can be activated by reactive oxygen species (ROS) produced during inflammation and injury [8]. The mechanism of action for ROS-mediated MMP activation was demonstrated in vitro by Rajagoplan [9], which showed ROS can oxidize a thiol bond responsible for activating MMP-2 and MMP-9. The information mentioned above indicates that ROS scavenger may inhibit the activation of MMPs.

Procyanidins are potent and safe natural product usually extracted from grape seed, with anti-oxidant, anti-inflammatory, and anti-allergic activities [10–12]. Basic and clinical studies show that procyanidins are health-protective agents and are beneficial to chronic venous insufficiency and diabetes [13–16]. Epidemiological studies indicated that populations consuming procyanidin-rich foods had lower incidences of inflammatory diseases, including metabolic syndrome and atherosclerosis [17, 18]. There is a large number of scientific evidence manifesting the inhibitory effects of procyanidins on inflammation [19]. It has been found that PC is even more potent scavenger of ROS as compared to known antioxidants, such as vitamins C and E, indicating that PC may be a potential therapeutic agent that targets MMP-9, and attenuates CCI-induced neuropathic pain.

We here provide the first evidences that procyanidins, safe and effective natural product, alleviate CCI-induced neuropathic pain via the inhibition of MMP-9/2 and the maturation of IL-1β.

## Methods

### Ethics statement

All procedures were strictly performed in accordance with the regulations of the ethics committee of the International Association for the Study of Pain and the Guide for the Care and Use of Laboratory Animals (The Ministry of Science and Technology of China, 2006). All animal experiments were approved by Nanjing Medical University Animal Care and Use Committee and were designed to minimize suffering and the number of animals used.

### Animals and neuropathic pain model

Adult male CD-1 mice (18–20 g wt) were provided by the Experimental Animal Center at Nanjing Medical University, Nanjing, China. Animals were housed five to six per cage under pathogen-free conditions with soft bedding under controlled temperature (22 ± 2 °C) and photoperiods (12:12-h light–dark cycle). The animals were allowed to acclimate to these conditions for at least 2 days before starting experiments. Animals were randomly divided into groups (*n* = 8). The sample size was designed on prior experience [20] and to be limited to the minimal as scientifically justified. For each group of experiments, the animals were matched by age and body weight. All surgeries were done under anesthesia induced by chloral hydrate (300 mg/kg, i.p.). Peripheral nerve injury was imitated by the model of chronic constriction injury of the sciatic nerve (CCI). In brief, the left common sciatic nerve of each mouse was exposed at the mid-thigh level. Proximal to the sciatic nerve’s trifurcation, nerve was separated from adhering tissue and 4 ligatures (5-0 chronic gut) were tied loosely around it with about 1 mm between ligatures. After surgery, the skin layers and muscle were sutured, and the surgery area was sterilized with iodine [21].

### Drugs and reagents

Procyanidins were purchased from Zelang Pharmaceutical Co. Ltd. (Nanjing, China) and were administrated via oral gavage. The purity of procyanidins was more than 95%. Procyanidins contained 1.1% monomeric, 34.2% dimeric, 24.9% trimeric, 6.7% tetrameric (totally 66.9% oligomeric procyanidins), and 33.1% polymeric procyanidins. Antibody for glyceraldehyde 3-phosphate dehydrogenase (GAPDH) was from Sigma (St. Louis, MO). Antibodies for ionized calcium-binding adapter molecule 1 (IBA-1), glial fibrillary acidic protein (GFAP), phosphorylated NR1 subunit (Ser896), phosphorylated PKC (pan) (gamma Thr514), phosphorylated p38 (Tyr182), and p65/RelA were from Cell Signaling Technology (Beverly, MA). Secondary antibodies were from Cell Signaling Technology (Beverly, MA, USA). Polyclonal antibody for IL-1β was from Santa Cruz (Dallas, TX). Lipopolysaccharide (LPS), rotenone, and dimethyl sulfoxide (DMSO) were purchased from Sigma-Aldrich (St. Louis, MO, USA). Fetal bovine serum (FBS) was purchased from Gibco, and other cell culture media and supplements were purchased from HyClone (Logan, UT, USA). Novex zymogram developing buffer was purchased from Invitrogen (Carlsbad, CA, USA). 3-(4,5-Dimethyl-2-thiazolyl)-2,5-diphenyl-2H-tetrazolium bromide (MTT) was purchased from Sunshine Biotechnology (Nanjing, China). MitoSOX was purchased from Thermo Fisher Scientific (Waltham, MA, USA). All other reagents were from Sigma-Aldrich (St. Louis, MO, USA).

### Gelatin zymography

Animals were killed by an overdose of chloral hydrate (300 mg/kg, i.p.), and spinal cord segments were rapidly dissected and homogenized in 1% NP40 lysis. Three hundred to 500 μg of protein per lane was loaded into the wells of precast gels (8% polyacrylamide gels containing 0.1% gelatin). After electrophoresis, each gel was incubated with 50 ml of zymogram developing buffer for 48 h (37.5 °C) in shaking bath. Then, the gels were stained with coomassie brilliant blue (1%, with 10% acetic acid, 10% isopropyl alcohol, diluted with dd H_2_O).

### Western blotting

The spinal cord segments at L1-L6 were rapidly removed and homogenized in RIPA Lysis Buffer after the animals deep anesthesia with chloral hydrate [22]. The protein concentrations were determined by BCA Protein Assay (Thermo Fisher, Waltham, MA), and 40 μg of proteins (unless specified otherwise) was loaded and separated by SDS-PAGE and electrophoretically transferred onto polyvinylidene fluoride membranes (Millipore Corp., Bedford, MA). The membranes were blocked with 5% bovine serum albumin for 1 h at room temperature, probed with antibodies overnight at 4 °C with the primary antibodies, and then incubated with HRP-coupled secondary antibodies. The primary antibodies used included IL-1β (1:500), p-NR1(1:1000), p-PKCγ (1:1000), p-p38 (1:1000), and IBA-1 (1:1000). For loading control, the blots were probed with antibody for GAPDH (1:8000). The filters were then developed by enhanced chemiluminescence reagents (PerkinElmer) with secondary antibodies (anti-rabbit or anti-mouse or anti-goat antibody, 1:1000) (Chemicon). Data were analyzed with the Molecular Imager (Gel DocTM XR, 170-8170) and the associated software Quantity One-4.6.5 (Bio-Rad Laboratories, Berkeley, CA).

### Behavioral analysis

Animals were habituated to the testing environment daily for at least 2 days before baseline testing. Mechanical sensitivity was detected by Von Frey Hairs (Woodland Hills, Los Angeles) test. Animals were placed in boxes set on an elevated metal mesh floor and were allowed 30 min for habituation before testing. The plantar surface of each hind paw was stimulated with a series of von Frey hairs with logarithmically incrementing stiffness perpendicularly to the plantar surface. Each mouse was tested for three times and the average of the threshold was measured.

### Immunofluorescence

After deep anesthesia by intraperitoneal injection of chloral hydrate (300 mg/kg), the animal was perfused transcardially with normal saline followed by 4% paraformaldehyde in 0.1 M PB (NaH_2_PO_4_ 2.964 g and Na_2_HPO_4_∙12H_2_O 28.998 g dissolve in deionized water to make 1 L solution), pH 7.4, each for 20 min. Then, L4 and/or L5 lumbar segment was dissected out and post-fixed in 4% paraformaldehyde. The embedded blocks were sectioned as 25 μm thick. Sections from each group (five mice in each group) were incubated with rabbit antibodies for IBA-1 (1:100) 12 h at 4 °C. Then, the free-floating sections were washed with PBS and incubated with the secondary antibody (1:300) for 2 h. After washing out three times with PBS, the samples were studied under an immunofluorescence microscope (Zeiss AX10, Germany) for morphologic details of the immunofluorescence staining. Examination was blindly carried out. Images were randomly coded, and the fluorescence intensities were analyzed by Image Pro Plus 6.0 software (Media Cybernetics Inc. Rockville, MD). The average green fluorescence intensity of each pixel was normalized to the background intensity in the same image.

### Cell preparation and stimulation

BV-2 cells were maintained in humidified 5% CO_2_ at 37 °C in Dulbecco’s modified Eagle’s Medium (DMEM) supplemented with 10% (*v*/*v*) FBS, penicillin (100 U/ml), and streptomycin (100 U/ml). For inducing inflammasome activation, 10^5^ cells were plated in 6-well plate overnight and the medium were changed to serum-free medium in the following morning, and then, the cells were treated with LPS (1 μg/ml) with or without procyanidins (1 ‰ DMSO) for 12 h. We performed the DMSO-only as the control. Cell extracts and precipitated supernatants were analyzed by immunoblotting.

### NF-κB activation assay

BV-2 cells were plated in class bottom cell culture dishes and treated with LPS (1 μg/ml) for 4 h with or without procyanidins (100 μM). Then, BV-2 cells were fixed with ice-cold methanol and were permeabilized with 0.25% Triton X-100/PBST. After blocking with 1% bovine serum albumin (BSA) in PBST for 1 h, the coverslips with BV-2 cells were incubated for 2 h at room temperature with the p65/RelA antibody diluted in 1% BSA (1:50). Then, the coverslips were exposed to the fluorescein isothiocyanate (FITC)-conjugated anti-rabbit IgG (1:100, at room temperature for 1 h) and then were rinsed three times with PBS. Finally, the coverslips were stained with 1 μg/mL DAPI (4′,6-diamidino-2-phenylindole, a fluorescent DNA dye to mark nucleus) for 1 min. Confocal microscopy analyze was carried out using Olympus FV1000 confocal system.

### Statistical analyses

SPSS Rel 15 (SPSS Inc., Chicago, IL) was used to conduct all the statistical analyses. Alteration of expression of the proteins detected and the behavioral responses were tested with one-way ANOVA and the differences in latency over time among groups were tested with two-way ANOVA, respectively. Bonferroni post hoc tests were conducted for all ANOVA models. Results are expressed as mean ± s.e.m. of three independent experiments. Results described as significant are based on a criterion of *P* < 0.05.

## Results

### PC directly inhibited the activity of MMP-9 and MMP-2 in vitro

**Fig. 1:**
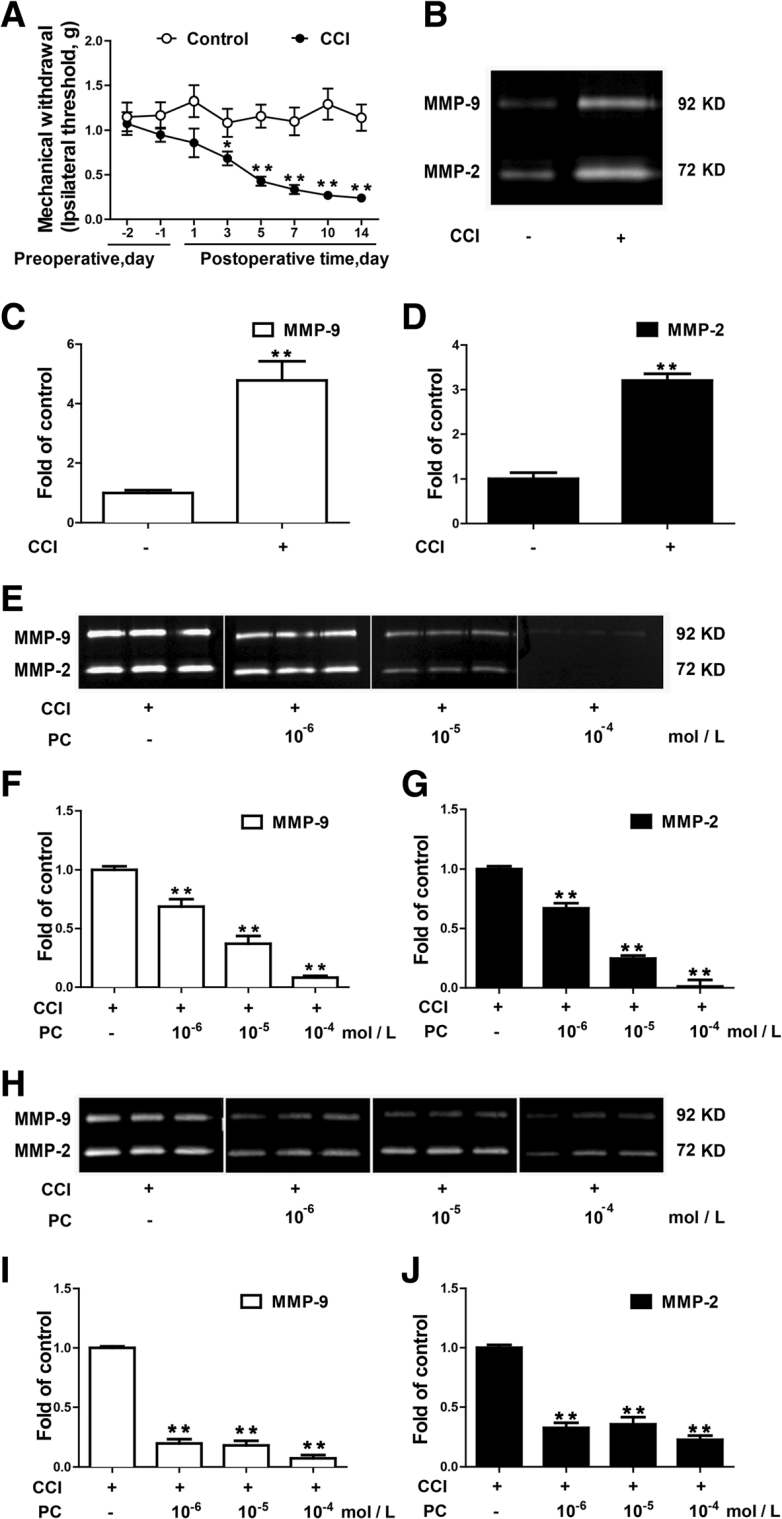
PC directly inhibited the chronic constrictive injury (CCI)-induced activation of matrix metalloproteinase (MMP)-9 and MMP-2. **a** Mechanical allodynia was significantly induced after CCI surgery. Mechanical pain threshold was tested by Von Frey Hairs. **b**–**d** MMP-9 and MMP-2 activity were significantly increased at 14 days after CCI injury. The lumbar spinal cords (L1–L6) were collected and analyzed 14 days after the CCI operation. Gelatin zymography was performed to determine MMPs’ activity. Representative bands and a data summary (*n* = 4) are shown. **e**–**g** PC directly reduced MMP-9 and MMP-2 activity of the spinal cord collected from CCI mice in vitro. The lumbar spinal cords (L1–L6) from mice treated CCI operation for 14 days were separated and homogenized. The same sample was loaded into each well of precast gels for gelatin zymography. After electrophoresis, the gels were cut into pieces. Each piece has three electrophoresis lanes on it and was then incubated in buffer with or without PC (10^−6^, 10^−5^, or 10^−4^ mol/L). Then, the gel pieces were stained with 1% Coomassie brilliant blue in the same box and captured in the same image. **h**–**j** PC directly reduced MMP-9 and MMP-2 activity of the spinal cord collected from normal mice in vitro. The same sample was loaded into each well of precast gels for gelatin zymography. After electrophoresis, the gel pieces were incubated in buffer with or without PC (10^−7^, 10^−6^, 10^−5^, or 10^−4^ mol/L). Significant difference was revealed following one-way ANOVA (*P < 0.05, **P < 0.01; Bonferroni post hoc tests)

Firstly, we measured mechanical threshold of CCI-treated mice by Von Frey Test. Fourteen days after CCI surgery, mechanical threshold was significantly reduced (Fig. 1a). We then evaluated CCI-induced MMP-9 and MMP-2 activation using gelatin zymography. MMP-9 and MMP-2 were significantly activated in the spinal cord of CCI mice (Fig. 1b–d). PC was administrated to evaluate its inhibition to the activity of MMP-9 and MMP-2 in vitro. Samples of spinal cords from normal (Fig. 1h–j) or CCI-treated mice (Fig. 1e–g) were loaded into each well for the electrophoresis. Then, the gel pieces loaded with proteins were incubated with PC with different concentrations. PC significantly inhibited the activity of MMP-9 and MMP-2 induced by CCI (Fig. 1e–g). At the same time, it inhibited the activity of MMP-9 and MMP-2 in basal condition (Fig. 1h–j).

### Single administration of PC attenuated CCI-induced neuropathic pain and suppressed CCI-induced activation of MMP-9 and MMP-2 in vivo

**Fig. 2:**
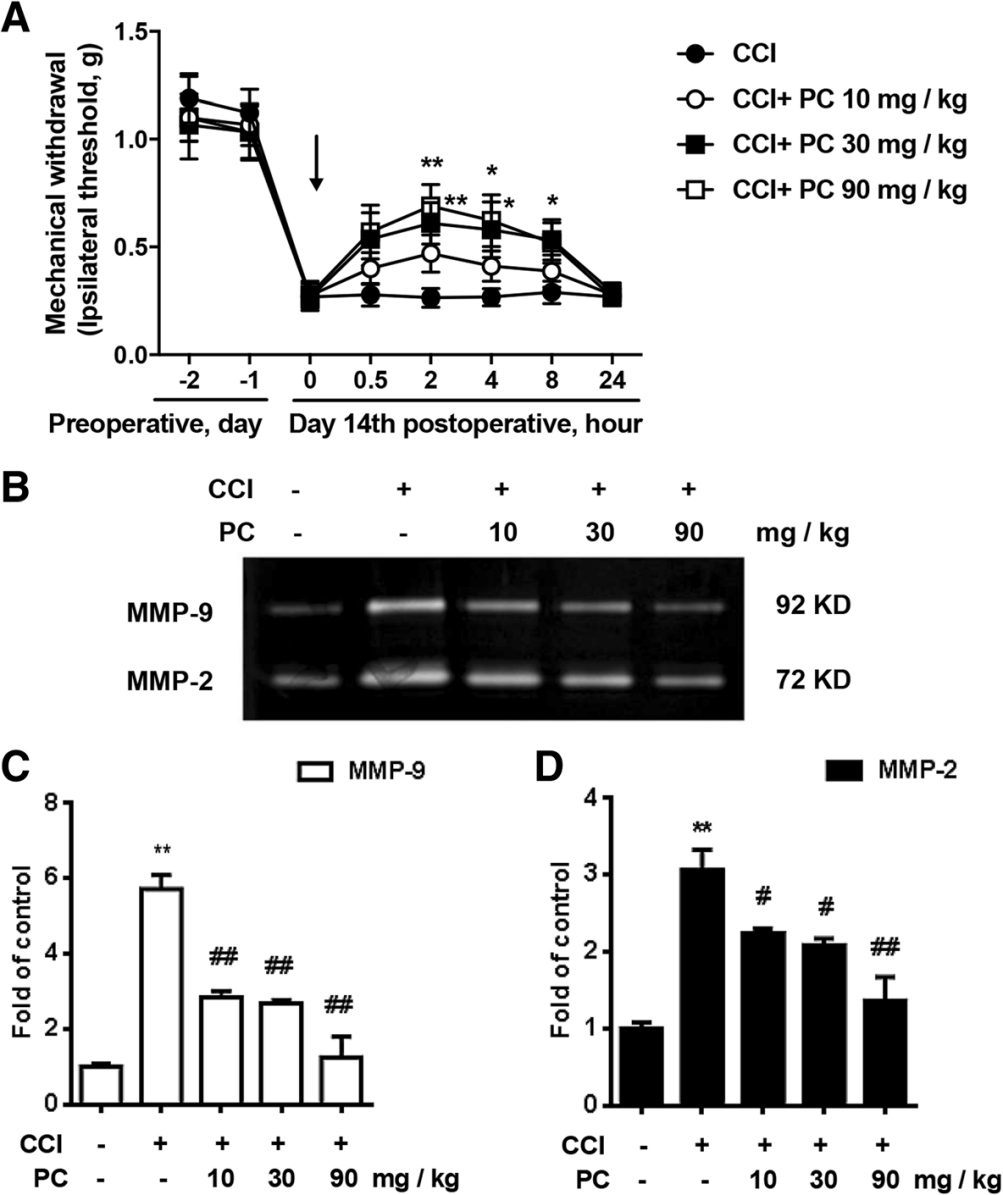
Single administration of PC attenuated CCI-induced neuropathic pain and suppressed CCI-induced activation of MMP-9/2 in vivo. **a** Single administration of PC (15, 30, 90 mg/kg, p.o.) at 14 days after CCI injury significantly attenuated CCI-induced mechanical allodynia (*n* = 6). **b**–**d** PC (15, 30, 90 mg/kg, p.o.) decreased the activity of MMP-9/2 in the spinal cords in a dose-dependent manner. The lumbar spines (L1–L6) were collected and analyzed 120 min after the drug administration. Representative bands and a data summary (*n* = 4) were shown. Significant difference was revealed following one-way or two-way ANOVA (**P < 0.01 vs. control; ^#^*P* < 0.05, ^##^*P* < 0.01 vs. CCI group; Bonferroni post hoc tests)

To investigate the effects of PC on MMP-9 and MMP-2 in vivo, single dosage of PC was given orally to CCI mice. Von Frey Hairs test was performed. After 14 days of CCI surgery, the mechanical threshold was marked decreased in CCI-treated mice. Pain-related behaviors were greatly ameliorated by PC (10, 30, and 90 mg/kg, p.o.) (Fig. 2a). Gelatin zymography results showed that PC inhibited the activity of MMP-9 and MMP-2 in the mouse spinal cords (Fig. 2b–d), which was reconcile with our data in vitro.

### Continuous administration of PC significantly alleviated CCI-induced neuropathic pain and suppressed CCI-induced activation of MMP-9 and MMP-2

**Fig. 3:**
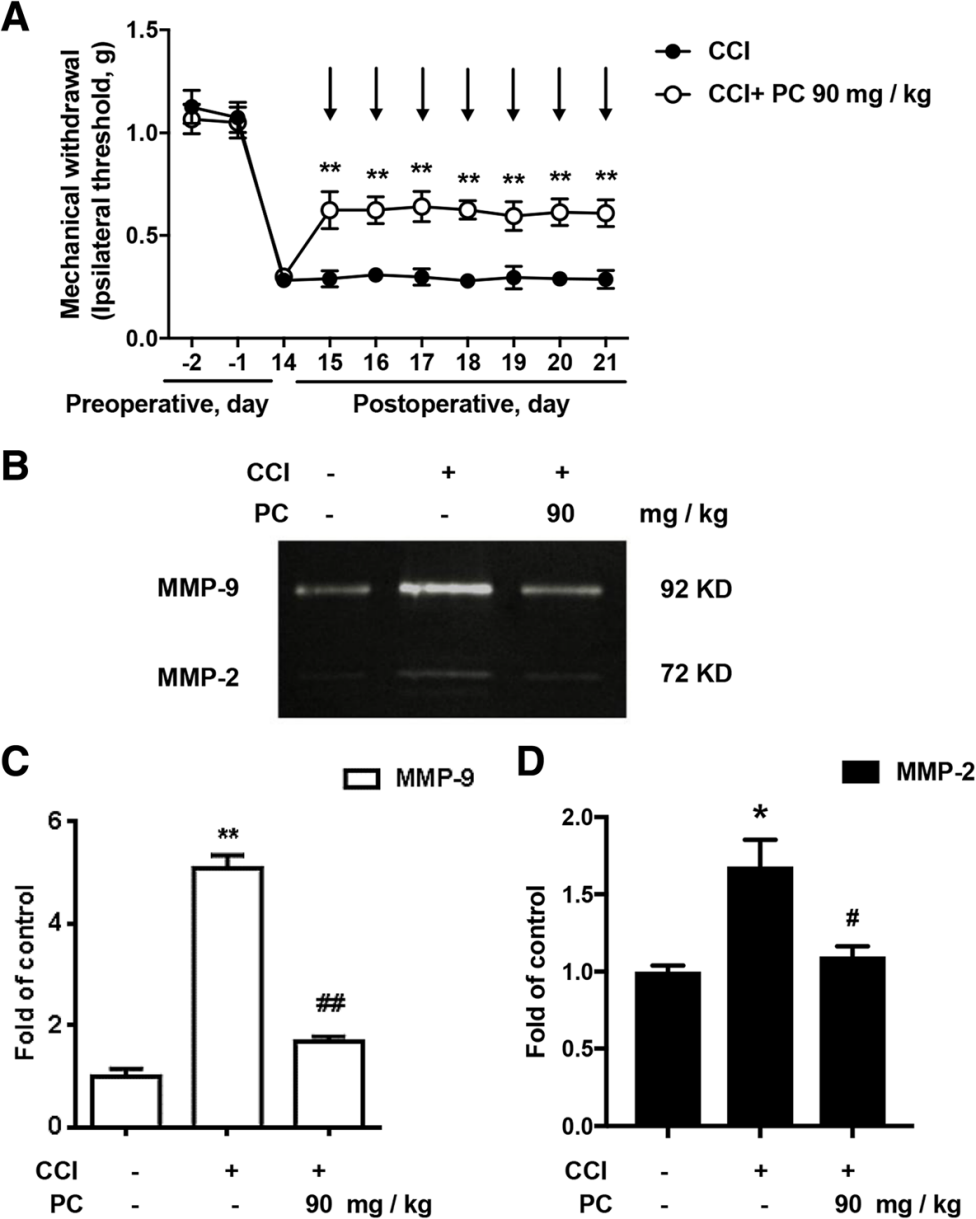
Consecutive administration of PC significantly alleviated CCI-induced neuropathic pain and suppressed CCI-induced activation of MMP-9/2 in vivo. (**a**) Consecutive administration of PC (90 mg/kg, p.o.) for 7 days significantly attenuated CCI-induced mechanical allodynia (*n* = 6). Behavior tests were carried out at 30 min after PC treatment. **b**–**d** PC (90 mg/kg, p.o.) decreased MMP-9/2′ activity in the spinal cords. The lumbar spines (L1–L6) were collected and analyzed 120 min after the last drug administration. Representative bands and a data summary (*n* = 4) were shown. Significant difference was revealed following one-way or two-way ANOVA (^**^P < 0.01 vs. control; ^#^*P* < 0.05, ^##^*P* < 0.01 vs. CCI group; Bonferroni post hoc tests)

Furthermore, the action of PC was studied by continuous administration after 14 days of CCI surgery. After continuous administration of PC (90 mg/kg, p.o.) for 7 days, the mechanical threshold was greatly ameliorated (Fig. 3a). In the meantime, PC significantly inhibited the activity of MMP-9 and MMP-2 in the spinal cord from CCI-treated mice in vivo (Fig. 3b, c).

### PC significantly inhibited CCI-induced IL-1β cleavage, PKCγ phosphorylation, and NR1 phosphorylation

**Fig. 4:**
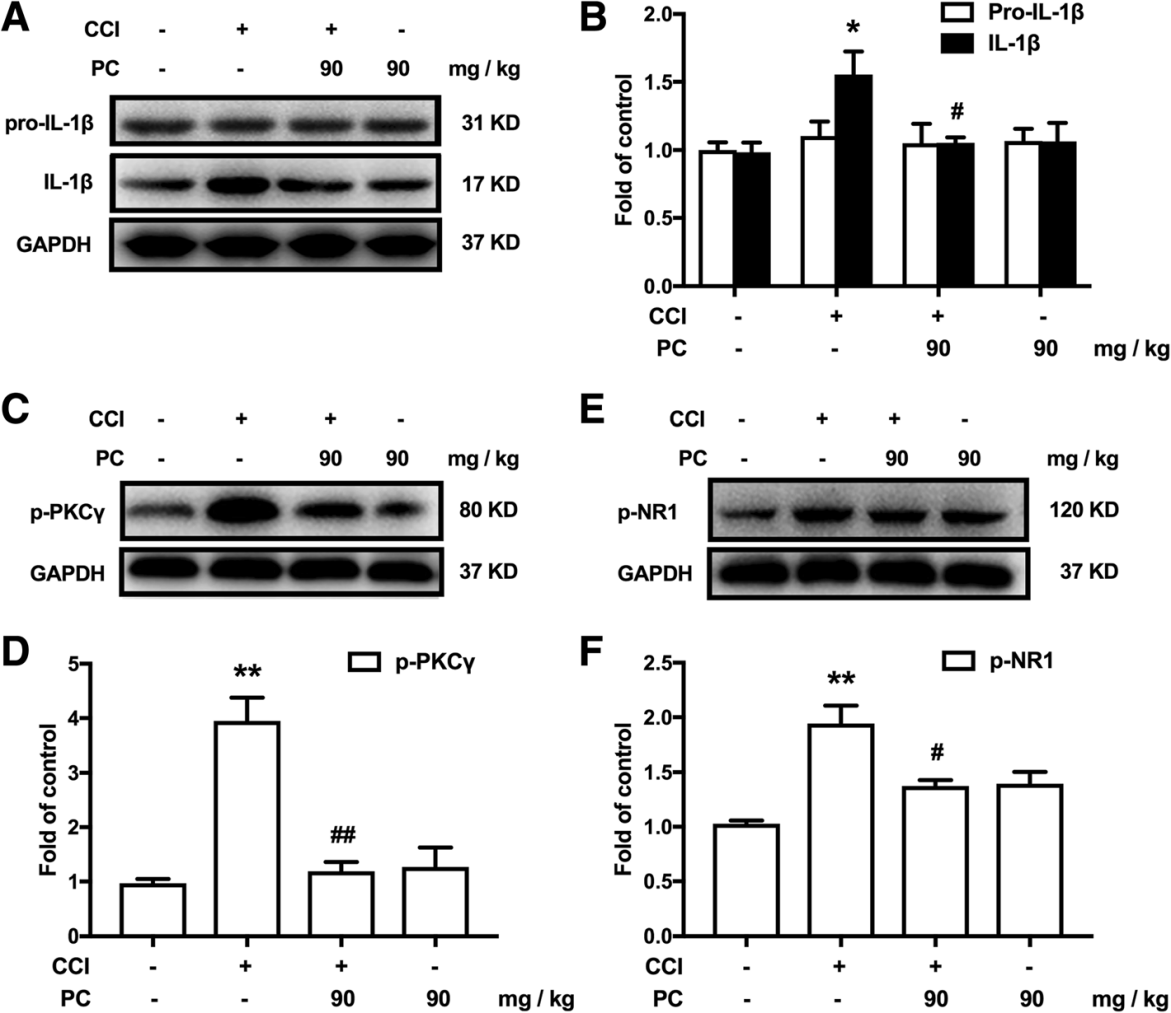
PC significantly attenuated CCI-induced neuron cells’ activation and interleukin (IL)-1β production in spinal cords. **a**, **b** Consecutive administration of PC significantly inhibited the cleavage of IL-1β but did not affect the production of pro-IL-1β. PC (90 mg/kg, p.o.) was administered daily from day 14 to day 20 after CCI operation. (80 μg of proteins were loaded when western blot was performed). **c**–**f** Single administration of PC inhibited CCI-induced phosphorylation of protein kinase C (PKC)γ and *N*-methyl-d-aspartate receptor (NR)1. PC (90 mg/kg, p.o.) was administered at day 14 after CCI operation. The lumbar spines (L1–L6) were collected and analyzed 120 min after the last administration. Significant difference was revealed following one-way or two-way ANOVA (^*^P < 0.05, ^**^P < 0.01 vs. control; ^#^*P* < 0.05, ^##^*P* < 0.01 vs. CCI group; Bonferroni post hoc tests)

A critical substrate of MMP-9 is IL-1β, which is essential for pain generation. Here, we reported that continuous administration of PC decreased the production of maturated IL-1β induced by CCI, while there are no significant changes on the level of pro-IL-1β after PC administration in CCI mice (Fig. 4a, b).

Studies show that IL-1β could stimulate neuron and glia via activating IL-1 receptor and subsequently results in the phosphorylation of PKCγ and NMDA [3], promoting the development of neuropathic pain. Thus, we investigated the role of PC on CCI-induced activation of neuron. Western blot analysis revealed that PC dramatically decreased CCI-induced phosphorylation of PKCγ and NR1 after continuous administration (90 mg/kg, p.o.) (Fig. 4c–f).

### PC significantly inhibited CCI-induced microglia activation

**Fig. 5:**
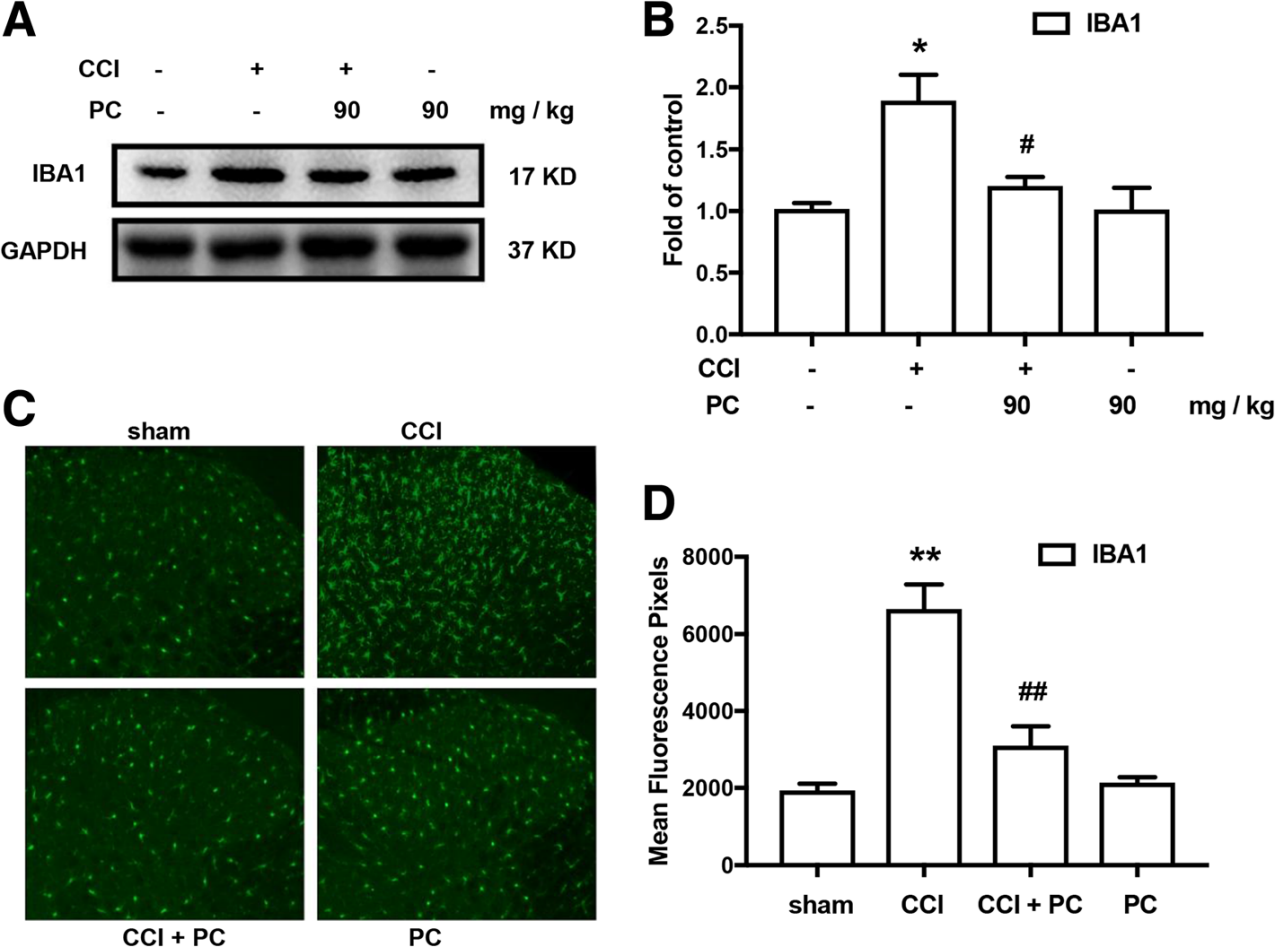
PC significantly inhibited CCI-induced activation of microglia marker IBA-1. **a**, **b** PC (90 mg/kg, p.o.) significantly decreased the expression of IBA-1 in the spinal cords. **c**, **d** Confocal images and immunofluorescence analysis data showing IBA-1 in the dorsal horns. PC (90 mg/kg, p.o.) significantly decreased IBA-1 expression in the spinal cords. Quantification of immunofluorescence was represented as mean fluorescence pixels in the superficial dorsal horns (*n* = 3, 5 images per animal). PC (90 mg/kg, p.o.) was consecutively administrated daily from day 14 to day 20 after CCI operation. The lumbar spines (L1–L6) were collected and analyzed 120 min after the last drug administration. Significant difference was revealed following one-way ANOVA (^**^P < 0.01 vs. control; ^#^*P* < 0.05, ^##^*P* < 0.01 vs. CCI group; Bonferroni post hoc tests)

Activation of microglia and neuro-microglia interactions are emerging as key mechanisms underlying neuropathic pain. Accumulating evidence has implicated that amount of inflammatory factors (e.g., IL-1β and TNF-α), proteinase (e.g., MMPs), and chemokines (CCL2) [7, 23, 24] are synthesized and released once microglia are activated, which further enhance neuronal signaling [25]. This crosstalk between microglia and neurons causes central sensitization and aggravates neuropathic pain. Therefore, we evaluated if PC could affect the activation of microglia induced by CCI. Our results showed that PC (90 mg/kg, p.o.) could significantly suppress the upregulation of microglia marker IBA-1 in spinal cord after CCI (Fig. 5a, b). Immunofluorescence of IBA1 in dorsal horn showed PC inhibited the activation of microglia (Fig. 5c, d).

### PC suppressed LPS-induced microglia activation by inhibiting p38 MAPK and NF-κB signaling

**Fig. 6:**
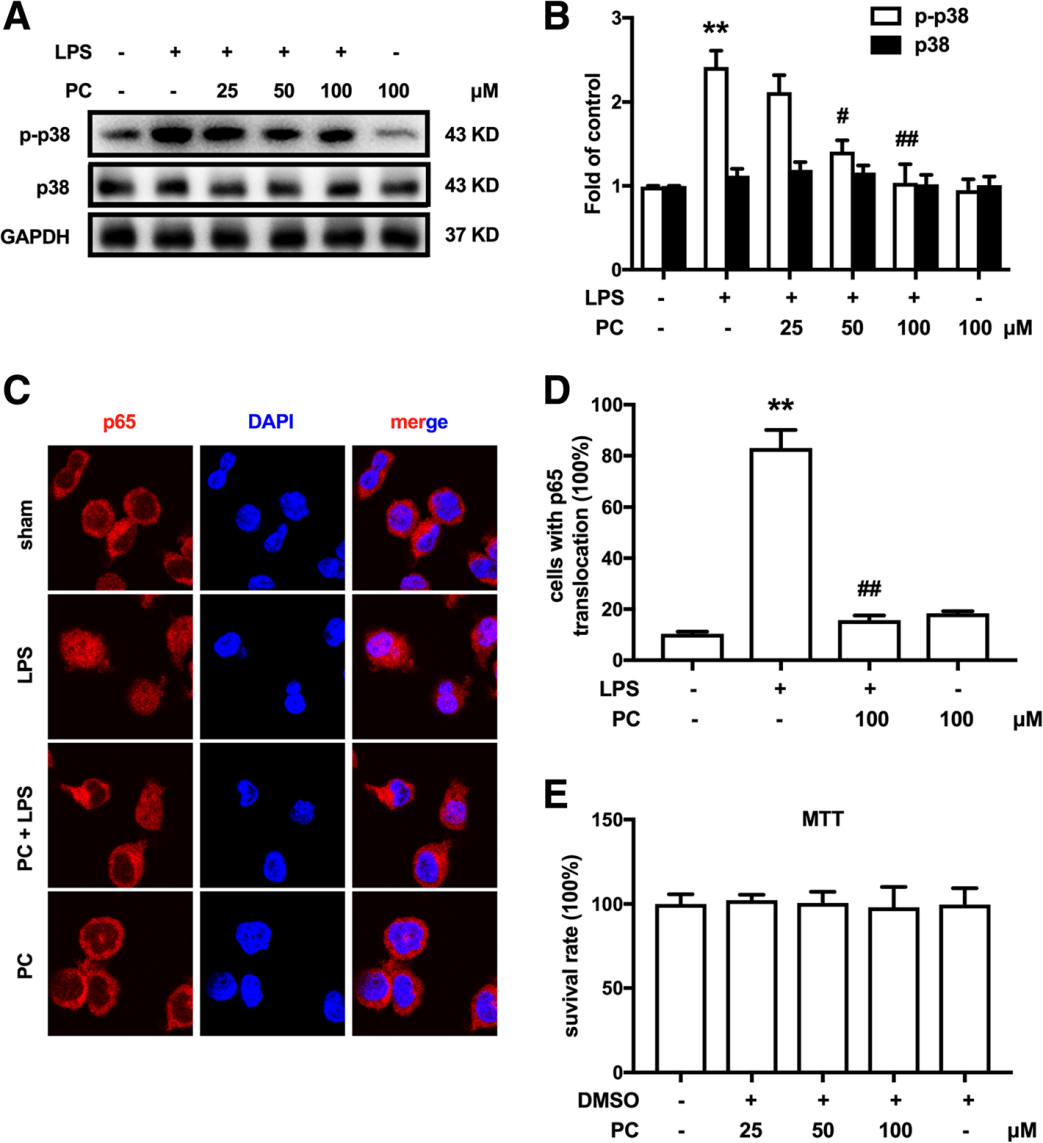
PC suppressed LPS-induced microglia activation by inhibiting p38 MAPK/NF-κB. **a**, **b** Pre-administration (15 min) of PC (25, 50, 100 μM) significantly inhibited the phosphorylation of p38 MAPK after LPS treatment for 2 h in a dose-dependent manner. Representative western blot bands and a data summary (*n* = 4) for p-p38 and p38 are shown. **c**, **d** PC (100 μM) inhibited the NF-κB translocation from the cytosol to the nucleus after LPS treatment for 4 h in BV-2 cells. **e** MTT experiments showed that different doses of procyanidins did not affect cell proliferation. Significant difference was revealed following one-way or two-way ANOVA (^*^P < 0.05, ^**^P < 0.01 vs. control; ^#^*P* < 0.05, ^##^*P* < 0.01 vs. LPS-treated group; Bonferroni post hoc tests; scale bar 10 μm)

The p38 MAPK phosphorylation and p65 NF-κB translocation from the cytoplasm to the nucleus were shown to promote the expression of MMP-9 in microglia [25]. Microglia BV-2 cells were used to further confirm the role of PC on p38 and NF-κB. As shown in Fig. 6a, c, LPS increased the phosphorylation of p38 MAPK and promoted the NF-κB translocation from the cytosol to the nucleus. Compared with the LPS-treated group, pre-administration (15 min) with PC significantly reduced p38 MAPK phosphorylation and p65 translocation (Fig. 6a–d). MTT assay indicated that the different doses of PC did not affect cell proliferation (Fig. 6e).

## Discussion

In this study, our major findings are as follows: (1) PC directly suppressed the activation of MMP-9 and MMP-2 in vitro and in vivo. (2) PC significantly attenuated the development of CCI-induced mechanical allodynia in mice. (3) PC markedly inhibited CCI-induced phosphorylation of PKCγ and NR1 and cleavage of IL-1β in spinal cords. (4) PC obviously inhibited CCI-induced microglia activation. (5) PC also inhibited LPS-induced p38 MAPK and NF-κB signaling in BV2 cells.

The process of MMP activation is called the “cysteine switch” whereby a conformational change occurs making the catalytic zinc accessible for a hydrolytic water molecule and for the substrate [26]. The “cysteine switch” is activated by oxidants, disulfides, alkylating reagents or Hg (II), and Au (I) compounds [27]. MMP activation also involves *S*-nitrosylation in vivo [28]. These reagents refer to lots of cytokines that are closely related to neuropathic pain, such as ROS and nNOS which produces ^−^ONOO-. As been proved, transcription of MMP-9 and MMP-2 relies on AP-1 or NF-κB pathway [29–32]. ROS is a vital stimulating factor in activating AP-1 and NF-κB and contributes to activation of MAPK/NF-κB signaling pathway [33, 34]. According the abovementioned research, ROS oxidizes a thiol bond to activate MMP-2 and MMP-9 in vitro. Recent researches demonstrate that ROS upregulates MMP-9 expression via NF-κB transcription factor [35].

As a natural antioxidant, we found that PC (90 mg/kg, p.o.) significantly decreased MDA level induced by CCI injury in the spinal cord (Additional file 1: Figure S1C) and reduced the oxidative stress in vivo. Additionally, PC significantly reduced the activity of MMP-9/2 both in vitro and in vivo (Figs. 1e, h, 2b, and 3b). Moreover, PC inhibited LPS-induced p38 and NF-κB signaling pathway in microglia (Fig. 6a, c) and inhibited CCI-induced MAPK phosphorylation in the spinal cord in vivo (Additional file 1: Figure S1A). Therefore, PC may inhibit MMP-9/2 not only directly on protein activity but also at transcriptional level, both of which are beneficial to the treatment of neuropathic pain. The exact mechanisms require further study.

In accordance with the results of gelatin zymography above, behavior tests showed that PC attenuates CCI-induced neuropathic pain (Figs. 2a and 3a) effectively. These results indicated that PC treatment in mice has a stable effect and has no drug resistance, providing reference for clinical application of PC in neuropathic pain. However, it must be mentioned that we could not exclude that the analgesic effect of PC may be partly due to inhibition of ROS production. Dozens of studies suggest that reactive oxygen species (ROS) are critically involved in the generation of pain in various painful conditions, including neuropathic and inflammatory pain. Antinociceptive effects of vitamins E or vitamins C which are known as powerful antioxidants have been reported in earlier studies. Recent study demonstrates that Vit C+E treatment reduces p38 but not ERK (p42/p44) phosphorylation in tissue extracts from the spinal cord and DRGs of SNI-treated animals [36]. PC is even more potent scavenger of ROS than vitamin E or vitamin C. Therefore, PC attenuates CCI-induced neuropathic pain might partly via inhibition of ROS production.

In addition, PC, as safe and effective compounds, can be absorbed from the gastrointestinal tract when people eat certain fruits and vegetables. It was usually administrated orally, and the oral bioavailability of PC was about 3–4% [37], but it was a relatively safe drug with an oral LD50 value of over 4000 mg/kg. The optimal dose for the attenuation of neuropathic pain is 90 mg/kg in mice, which is exceedingly safe. Therefore, PC would be safe and effective for inhibiting neuropathic pain.

Central sensitization refers to the process through which a state of hyperexcitability is established in the central nervous system, leading to enhanced processing of nociceptive (pain) messages. Although numerous mechanisms have been implicated in central sensitization, the most important of which are hypersensitivity mediated by NMDA receptor and interaction between neuron and glia. After injury, increased release of neurotransmitters from nociceptors will sufficiently depolarize postsynaptic neurons to activate quiescent NMDA receptors. The consequent increase in calcium influx can phosphorylate downstream molecules (e.g., PKCγ), which in turn will exacerbate responses in spinal cord neurons [4, 38]. Previous studies showed that MMP-9 activates NR1 via integrin β1 or nitric oxide pathways. Our results showed that PC could significantly suppress phosphorylation level of NR1 and PKC (Fig. 4c–f). Meantime, glial cells, notably microglia, also contribute to the central sensitization process that occurs in the setting of injury. The activated microglia release a panoply of signaling molecules, including cytokines (such as TNF-a, IL-1β, and IL-6), which enhance neuronal central sensitization and nerve injury-induced persistent pain. Our results showed that PC could significantly inhibit the activation of microglia (Fig. 5). The results showed that PC could inhibit the downstream signals corresponding to MMP-9 activation.

Moreover, PC significantly reduced the phosphorylation of p38 MAPK and p65 translocation in BV-2 cells (Fig. 6a–d). Study shows that p38/NF-κB signaling pathway play an important role in the process of neuropathic pain [7]. Activation of most microglial receptors can converge on p38 phosphorylation, which leads to the synthesis and release of multiple inflammatory mediators, such as IL-1β [39]. As has been proved, MMP-9/2 contributed to cleavage of IL-1β [7]. Our data showed that the CCI-induced production of active IL-1β in spinal cord was significantly inhibited by PC (Fig. 4a, b), whereas PC had little influence on the level of pro-IL-1β, which indicated its effects on MMP-9/2 cleavage activity. Yet unfortunately, we did not investigate the source of MMP-9/2 for it is a little bit difficult to track the source in vivo. MMP-9/2 may be derived from neurons, microglia, and astroglia in the condition of neuropathic pain. Our previous study and other researches showed that neurons [40], microglia [40], and astroglia [40] could express MMPs in vitro following stimulation with IL-1β. On account of the widespread presence of MMP-9/2, it is especially important to decrease the activity of MMP-9/2 for easing the inflammation. Furthermore, previous research by our laboratory and others has demonstrated a role for other signaling pathways converging on NACHT, LRR, and PYD domain-containing protein 3 (NLRP3). NLRP3 is known for its role critical for caspase-1 cleavage and maturation. Caspase-1 is crucial for the processing of pro-IL-1β to mature IL-1β [41]. In this process, reactive oxygen species (ROS) generation is reported to induce unprompted NLRP3 inflammasome activation [42, 43]. Recent research by our laboratory proved that PC alleviates morphine tolerance by inhibiting activation of NLRP3 inflammasome in microglia, which mediated cleavage of IL-1β [44]. Therefore, PC may also inhibit IL-1β through NLRP3.

## Conclusions

In summary, our results demonstrated that PC attenuates CCI-induced neuropathic pain via inhibiting MMP-9/2 activity, maturated IL-1β production and microglia activation. Our study suggests that PC may be a potential drug candidate for neuropathic pain treatment.

## Additional file

Additional file 1: **Figure S1.** PC significantly inhibited CCI-induced phosphorylation of JNK, p38, and ERK and decreased p65 expression in nuclear in vivo. (A) Single administration of PC (90 mg/kg, p.o.) significantly decreased the expression of p-JNK, p-p38, and p-ERK (Cell Signaling Technology, MA, USA) in the spinal cord of CCI mice. (B) Single administration of PC (90 mg/kg, p.o.) significantly decreased the expression of p65 (Cell Signaling Technology, MA, USA) in nuclear in vivo. (C) Single administration of PC (90 mg/kg, p.o.) significantly decreased MDA level induced by CCI injury in the spinal cord. PC (90 mg/kg, p.o.) was administered at day 14 after CCI operation. The lumbar spines (L1–L6) were collected and analyzed 120 min after the last drug administration. Representative bands and a data summary (*n* = 4) was shown. (*P < 0.05, **P < 0.01 vs. control; ^#^*P* < 0.05, ^##^*P* < 0.01 vs. CCI group; Bonferroni post hoc tests). (DOCX 262 kb)

## Abbreviations

BSA: Bovine serum albumin
DMEM: Dulbecco’s modified Eagle’s medium
DMSO: Dimethyl sulfoxide
GAPDH: Glyceraldehyde-3-phosphate dehydrogenase
IL-1β: Interleukin-1β
MAPK: Mitogen-activated protein kinase
MMP-9/2: Matrix metalloproteinase-9/2
NMDA: *N*-Methyl-d-aspartic acid
NR1: *N*-Methyl-d-aspartate receptor 1
PBS: Phosphate-buffered saline
PC: Procyanidins
PKC: Protein kinase C
SDS-PAGE: Sodium dodecyl sulfate polyacrylamide gel electrophoresis
