# Supplementary material for: Procyanidins attenuate neuropathic pain by suppressing matrix metalloproteinase-9/2
Source: J Neuroinflammation. 2018 Jun 21;15:187. doi: 10.1186/s12974-018-1182-9 (PMC6013948; doi:10.1186/s12974-018-1182-9)
Supplement: Supplementary file 1 — Figure S1. PC significantly inhibited CCI-induced phosphorylation of JNK, p38, and ERK and decreased p65 expression in nuclear in vivo. (A) Single administration of PC (90 mg/kg, p.o.) significantly decreased the expression of p-JNK, p-p38, and p-ERK (Cell Signaling Technology, MA, USA) in the spinal cord of CCI mice. (B) Single administration of PC (90 mg/kg, p.o.) significantly decreased the expression of p65 (Cell Signaling Technology, MA, USA) in nuclear in vivo. (C) Single administration of PC (90 mg/kg, p.o.) significantly decreased MDA level induced by CCI injury in the spinal cord. PC (90 mg/kg, p.o.) was administered at day 14 after CCI operation. The lumbar spines (L1–L6) were collected and analyzed 120 min after the last drug administration. Representative bands and a data summary (n = 4) was shown. (*P < 0.05, **P < 0.01 vs. control; #P < 0.05, ##P < 0.01 vs. CCI group; Bonferroni post hoc tests). (DOCX 262 kb) [file 12974_2018_1182_MOESM1_ESM.docx]

**Procyanidins attenuates neuropathic pain by suppressing matrix** **metalloproteinases-9/2**

Cailong Pan^123†^, Chaoyu Wang^12†^, Li Zhang^5†^, Ling Song^12^, Yuan Chen^2^, Bingqian Liu^6^, Wen-Tao Liu^12^, Liang Hu^12*^, Yinbing Pan^4*^

^†^ Authors equally contribute to this work

^*^ Addressed correspondence to lianghu@njmu.edu.cn, Neuroprotective Drug Discovery Key Laboratory of Nanjing Medical University, Department of Pharmacology, Nanjing Medical University, Nanjing 210029, China, or panyinbing@sina.com, Department of Anesthesiology, The First Affiliated Hospital of Nanjing Medical University, Nanjing 210029, China

Tel: +86-25-86862127

co-authors' email addresses

Cailong Pan: 651612325@qq.com; Chaoyu Wang: 1015217627@qq.com, Li Zhang: drzhangli@njmu.edu.cn, Ling Song: 1716065690@qq.com, Yuan Chen: 724996792@qq.com, Wen-Tao Liu: painresearch@njmu.edu.cn, Yinbing Pan: panyinbing@sina.com, Liang Hu: lianghu@njmu.edu.cn

**Supplementary material and methods**

**Determination of lipid peroxidation in the spinal cord.** The homogenate was centrifuged at 1,000 g at 4ºC for 10 min after the lumbar spinal cord (L1-L6) was washed in PBS and homogenized with the IKA homogenizer (IKA-WERKE, Germany) (15 s × 3) in 1 ml of buffer (mM: NaCl 136, surose 10, Tris-HCl 10, EDTA 1, pH 7.4). The 10% homogenate supernatant was mixed with 15% trichloroacetic acid and 0.375% thiobarbituric acid. 0.01% butylated hydroxytoluene was added to the mixture to prevent autoxidation of the sample, and the mixture was heated at 100°C for 15 min. After being cooled off, the mixture was centrifuged at 1,000 g for 20 min, then the absorbance of the supernatant was measured by a spectrophotometer at 532 nm, and results were expressed as nmol per mg protein.

**Preparation of nuclear and cytoplasmic extracts.** The compartment protein extraction kit (Jiancheng Institute of Biotechnology,Nanjing, China) was used to prepare nuclear and cytoplasmic extracts from the lumbar spinal cords (L1-L6). The procedure was performed as recommended by the manufacturer. Briefly, each tissue was homogenized in lysis buffer C containing protease inhibitor cocktails, and after centrifugation at 5000g at 4°C for 10min the supernatant was retained as the cytosolic fraction. The nuclear pellet was washed twice, resuspended in lysis buffer N containing protease inhibitor cocktails, and after centrifugation at 5000g at 4°C for 20min the supernatant was retained as the nuclear

**Supplementary figure**

**
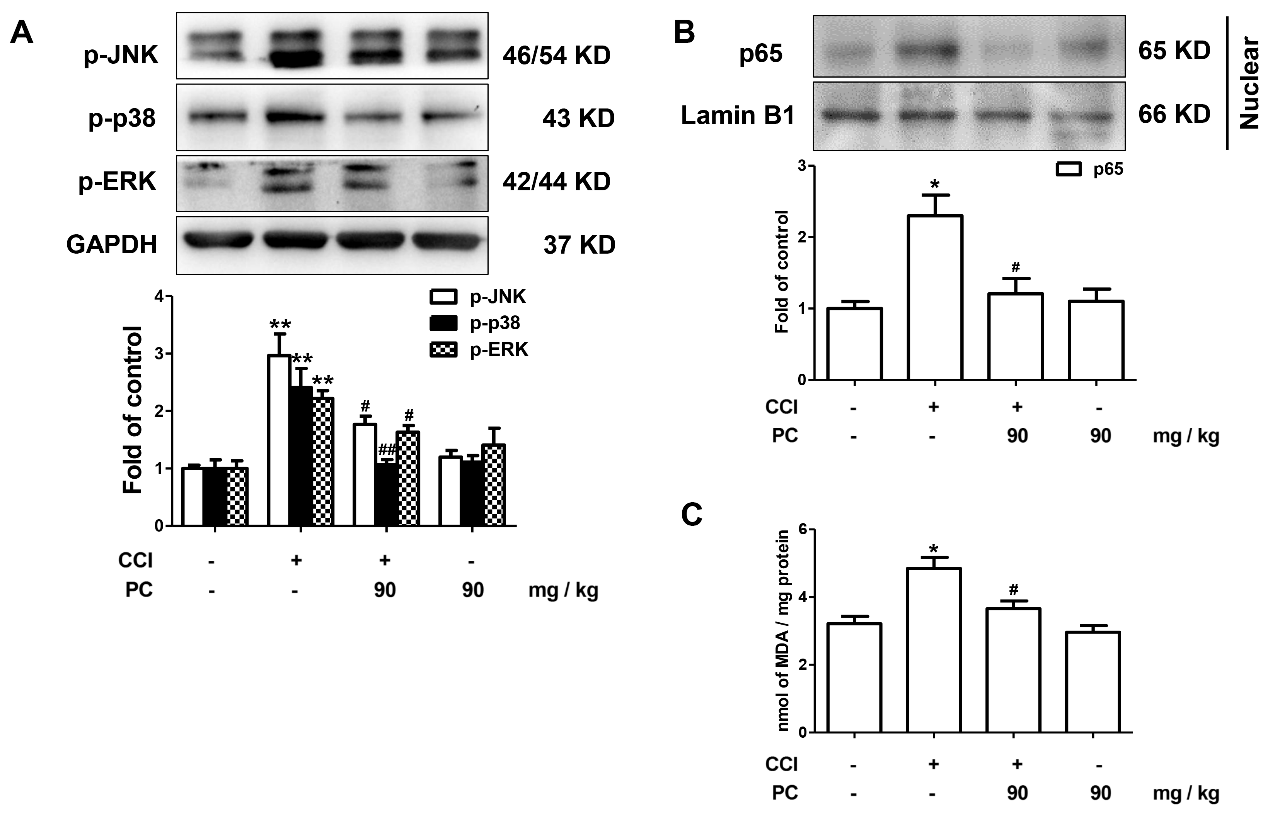
**

**Figure S1** PC significantly inhibited CCI induced phosphorylation of JNK, p38, ERK and decreased p65 expression in nuclear in vivo. (A) Single administration of PC (90 mg / kg, p.o.) significantly decreased the expression of p-JNK, p-p38 and p-ERK (Cell Signaling Technology, MA, USA) in the spinal cord of CCI mice. (B) Single administration of PC (90 mg / kg, p.o.) significantly decreased the expression of p65 (Cell Signaling Technology, MA, USA) in nuclear in vivo. (C) Single administration of PC (90 mg / kg, p.o.) significantly decreased MDA level induced by CCI injury in the spinal cord. PC (90 mg / kg, p.o.) was administered at day 14 after CCI operation. The lumbar spines (L1–L6) were collected and analysed 120 min after the last drug administration. Representative bands and a data summary (n=4) was shown. (^*^*P* < 0.05, ^**^*P* < 0.01 *vs*. control; ^#^*P* < 0.05, ^##^*P* < 0.01 *vs*. CCI group; Bonferroni post hoc tests)
